# Vascular endothelial function of patients with stable coronary artery disease

**DOI:** 10.12669/pjms.313.6892

**Published:** 2015

**Authors:** Zhe Wang, Xinchun Yang, Jun Cai, Hui Shi, Guangzhen Zhong, Hongjie Chi

**Affiliations:** 1 Zhe Wang, MD. Heart Center, Beijing Chaoyang Hospital, Capital Medical University, Beijing 100020, China; 2 Xinchun Yang, MD. Heart Center, Beijing Chaoyang Hospital, Capital Medical University, Beijing 100020, China; 3 Jun Cai, MD. Heart Center, Beijing Chaoyang Hospital, Capital Medical University, Beijing 100020, China; 4 Hui Shi, MD. Heart Center, Beijing Chaoyang Hospital, Capital Medical University, Beijing 100020, China; 5 Guangzhen Zhong, MD, Heart Center, Beijing Chaoyang Hospital, Capital Medical University, Beijing 100020, China; 6 Hongjie Chi, MD. Heart Center, Beijing Chaoyang Hospital, Capital Medical University, Beijing 100020, China

**Keywords:** Atherosclerotic coronary heart disease, Endothelial dysfunction, Reactive hyperemia index

## Abstract

**Objectives:**

To evaluate vascular endothelial function and contributing factors in coronary heart disease (CHD) patients.

**Methods:**

One hundred twenty six CHD outpatients were randomly recruited. Reactive hyperemia index (RHI) <1.67 indicates endothelial dysfunction. Correlation between RHI and different biochemical parameters was evaluated.

**Results:**

RHI in patients receiving statins treatment was significantly higher than patients without statins treatment (P<0.05). RHI in patients with more than 3 risk factors for CHD was also markedly lower than that in patients with ≤2 risk factors (P<0.05). Patients with lesions at several branches of coronary artery had a markedly lower RHI when compared with those with coronary lesions at a single branch (P<0.05). For patients without statins treatment, RHI increased significantly after statins treatment for 1 month (P=0.01). In patients with endothelial dysfunction, FBG, HbA1C, hs-CRP and Hcy were significantly higher than those in patients with normal endothelial function (P<0.05 for all). Smokers with CHD had a remarkably lower RHI when compared with non-smokers (P<0.05).

**Conclusions:**

Smoking, FBG, HbA1C, Hcy and hs-CRP are significantly associated with endothelial dysfunction. Endothelial dysfunction is also related to the numbers of risk factors for CHD, degree of coronary lesions and statins. Statins treatment may significantly improve the endothelial function of CHD patients.

## INTRODUCTION

The recent studies have shown that the vascular endothelial dysfunction plays an important role in the pathogenesis of ischemic heart diseases. Endothelial dysfunction is closely associated with the occurrence and development of a variety of diseases, and some diseases may present with manifestations of endothelial dysfunction at early stage.^1^-^3^ Endothelial dysfunction is an initiator and a key step in the pathogenesis of atherosclerosis and may promote the progression of atherosclerosis into severe cardiovascular endpoint events. There is evidence^4^ that acute attack of coronary ischemia has a poor relationship with the degree of coronary stenosis, but is closely related to the endothelial dysfunction of coronary artery. Coronary angiography shows acute attack of coronary ischemia has a poor relationship with the degree of coronary stenosis, but is closely related to the endothelial dysfunction of coronary artery.

Thus, early detection of endothelial dysfunction is helpful to identify subjects who seem to be healthy and have high risk for cardiovascular diseases due to unhealthy lifestyle and to predict the risk for severe cardiovascular events in patients with coronary heart diseases (CHD). In recent years, increasing clinicians pay attention to the measurement of endothelial function.

## METHODS

To explore the relationship between CHD and vascular endothelial function, CHD outpatients were recruited from our hospital and received the non-invasive detection of endothelial function (reactive hyperemia index, RHI) with Endo-PAT2000 Diagnostic System. Then, the correlation of number of risk factors for CHD, degree of coronary lesions and use of statins with endothelial function was evaluated.

### Clinical information

A total of 126 outpatients with stable CHD were randomly recruited from April 2013 to December 2013 from our hospital. There were 71 males (56.3%) and 55 females (43.7%). The mean age was 56±12.9 years (range: 22-84 years). Coronary angiography showed ≥50% coronary stenosis.

### Exclusion criteria

heart failure (EF<50%), cardiomyopathy, severe valvular heart disease, immune disease, cerebrovascular diseases causing hemiplegia, homological diseases, severe liver disease, renal failure and cancers. The study protocol has been approved by the institute’s committee on human research of our hospital.

The general information was collected at baseline, and detection of RHI, Coronary angiography and detection of blood biochemistry were done in all the patients. RHI was measured with EndoPAT-2000 non-invasive Diagnostic System (Itamar, Israel) as an indicator of endothelial function in the morning at a fasting status and before medication. The endothelial function was evaluated on the basis of RHI, and the correlation of number of risk factors for CHD, degree of coronary lesions and use of statins with endothelial function was determined.

### Risk factors for CHD

Male, postmenopausal women, hypertension (blood pressure ≥ 140/90 mmHg or antihypertensive medications, diabetes (fasting glucose >7.1 mmol/L or medication with glucose-lowering drugs), hyperlipidemia, family history of CHD at early age and smoking (≥5 cigarette without smoking cessation).

### Conditions for RHI measurement

Patients were laid in a supine position in a dark room with appropriate temperature for 10 min, and fasted. On the day of measurement, exercise was avoided. Patients were asked to stop smoking, drinking coffee, discontinuation of long-acting nitrates, telephoning, medicating with high dose vitamin C or drinking fluid containing vitamin C and to keep calm at 12 h before measurement. If a patient developed acute diseases (such as flu), measurement were deferred.

**Diagnosis:**

### Diagnosis of atherosclerotic coronary heart disease

CHD was diagnosed by coronary angiography. Judkins method was employed to perform right and left coronary angiography to evaluate the degree of stenosis of the main, circumflex and anterior descending branches of the left coronary artery and the main and other branches of right coronary artery. The diameter was measured by visualization to determine the degree of stenosis, and CHD was diagnosed when the ≥50% stenosis was observed.

### Definition of hypertension

In the absence of pressure lowering drugs, blood pressure was measured thrice on different days. When the systolic blood pressure was ≥140 mmHg and/or the diastolic blood pressure was ≥90 mmHg (diagnostic criteria from WHO/ISH in 1999), hypertension was diagnosed.

### Diagnostic criteria for diabetes

Diabetes was diagnosed according to clinical manifestations and blood glucose (plasma glucose ≥ 11.1 mmol/L [200mg/dl] at any time point, or fasting plasma glucose ≥ 7.0 mmol/L [126 mg/dl], or plasma glucose ≥ 11.1 mmol/L [200mg/dl] at 2 h after oral glucose tolerance tests [OGTT]) according to the diagnostic criteria of WHO in 1999.

### Diagnostic criteria for dyslipidemia

Dyslipidemia was diagnosed when the total fasting serum cholesterol was ≥5.18 mmol/L, triglyceride was ≥1.70 mmol/L or low-density lipoprotein cholesterol was ≥3.37 mmol/L according to the Chinese Guideline for the Prevention and Treatment of Dyslipidemia (2007).

### Statistical analysis

Statistical analysis was performed with SPSS version 17.0. Quantitative data were expressed as mean ± standard deviation, and comparisons between two groups were done with independent t test. Qualitative data were compared with chi square test. A value of P<0.05 was considered statistically significant.

**Table-I:** General information of all the patients at baseline.

	RHI>0.67(n=92)	RHI≤0.67 (n=34)	P
Age (yrs)	60±10	62±11	P>0.05
Male sex, no. (%)	61(66.3%)	22(64.7%)	P>0.05
Body mass index (kg/m^2^)	24.1±4.3	24.5±5.2	P>0.05
Current smoking, no. (%)	11(12.0%)	12(35.3%)	P<0.05*
Hypertension, no. (%)	54(58.7%)	19(55.9%)	P>0.05
Diabetes, no. (%)	34(36.9%)	12(35.3%)	P>0.05
Dyslipidemia, no. (%)	42(45.7%)	14(41.2%)	P>0.05
Family history of CAD, no. (%)	49(53.3%)	18(52.9%)	P>0.05
Systolic BP, mmHg	129.1±18.6	127.2±13.7	P>0.05
Diastolic BP, mmHg	71.2±12.7	73.6±11.6	P>0.05
FBG (mmol/L)	5.89±0.84	6.75±0.56	P<0.01*
Hemoglobin A1c (%)	5.68±0.72	6.58±0.47	P<0.01*
Total cholesterol (mmol/L)	5.21±1.32	5.35±1.19	P>0.05
HDL cholesterol (mmol/L)	1.04±0.14	1.06±0.25	P>0.05
LDL cholesterol (mmol/L)	2.61±1.13	2.79±0.96	P>0.05
Triglycerides (mmol/L)	1.72±0.30	1.79±0.59	P>0.05
hs-CRP (mg/dl)	1.58±0.47	2.59±0.54	P<0.01*
Hcy (umol/L)	5.89±0.84	6.75±0.56	P<0.01*
Aspirin, no. (%)	87(94.6%)	31(91.2%)	P>0.05
CCB, no. (%)	33(35.9%)	11(32.4%)	P>0.05
ACE-I or ARB, no. (%)	62(67.4%)	21(61.8%)	P>0.05
Beta-blockers, no. (%)	75(85.9%)	27(79.4%)	P>0.05
Anti-diabetic drugs, no. (%)	31(33.7%)	11(32.4%)	P>0.05
HMG-CoA, no. (%)	82(89.1%)	14(41.1%)	P>0.05

* P<0.05 between two groups; RHI: reactive hyperemia index; CAD: coronary artery disease;

BP: blood pressure; FBG: fasting blood glucose; HDL: high-density lipoprotein;

LDL: low-density lipoprotein; hs-CRP: high-sensitivity C-reactive protein; Hcy: homocysteine;

CCB: calcium channel blocker; ACE-I: angiotensin converting enzyme inhibitors;

ARB: angiotensin receptor blocker; HMG: 3-hydroxy-3-methyl-glutaryl

**Table-II:** Correlation of RHI with use of statins, degree of coronary lesions and risk factors.

	n	RHI	P
*Use of statins*			
Yes	96	1.96±0.57	P<0.05*
No	30	1.61±0.71	
Before	26	1.69±0.13	P<0.05*
After	26	1.98±0.25	
*Coronary lesions*			
Several branches	87	1.59±0.57	P<0.05*
Single branch	39	1.81±0.68	
*Risk factors for CHD*			
≤2	58	1.98±0.87	P<0.05*
≥3	68	1.56±0.49	

* P<0.05 between two groups,

CHD: coronary artery disease.

## RESULTS

### General information

Of 126 patients with CHD, 92 (73%) patients had normal endothelial function, and 34 (27%) had endothelial dysfunction. Smokers had a significantly lower RHI when compared with non-smoker (P<0.05). The FBG, HbA1C, Hcy and hs-CRP in patients with endothelial dysfunction were significantly higher than those in patients with normal endothelial function (P<0.05 for all).

### Correlation of RHI with use of statins, degree of coronary lesions and number of risk factors

Of CHD patients, the RHI in patients with statins treatment was significantly higher than that in those without use of statins (1.96±0.57 vs. 1.61±0.71, P<0.05). Patients with 3 or more risk factors had a markedly higher RHI when compared with those having ≤2 risk factors (1.56±0.49 vs.1.98±0.87, P<0.05). In addition, the RHI in patients with lesions at several branches of coronary artery was dramatically lower than that in patients with lesions at a single branch (1.59±0.75vs. 1.81±0.6, P<0.05). Of 26 patients without use of statins (exclusion of 4 patients with elevated muscle enzymes and rash), RHI increased significantly after treatment with statins for 1 month when compared with that before therapy (P=0.01).

## DISCUSSION

Vascular endothelial cells refer to a single layer of cells covering on the inside wall of blood vessels. Endothelial cells are not only a permeability barrier, but an endocrine and paracrine organ with multiple functions. These cells can express and secret a lot of biologically active mediators playing important roles in regulating the vascular tone, thrombosis, growth of smooth muscle cells, immune response, extracellular matrix and inflammatory reaction. The normal endothelial function is a basis for the maintenance of cardiovascular stability. When the endothelial cells are repeatedly injured by blood flow, tobacco, drugs, high blood lipid, high blood glucose, high blood pressure, high blood homocysteine and low estrogen, endothelial dysfunction occurs, nitric oxide secretion decreases and endothelin secretion increases, resulting in atherosclerosis, hypertension, myocardial ischemia, cerebrovascular diseases and diabetes. Some diseases may present endothelial dysfunction at early stage.

Hamburg et al.^5^ conducted a study on 1957 subjects recruited from 3rd generation, and results showed Endo-PAT index was negatively associated with several risk factors of cardiovascular diseases (such as male gender, body mass index, total cholesterol/high-density lipoprotein, diabetes, smoking and lipid-lowering therapy). Kuvin et al.^6^ found Endo-PAT index was also negatively related to some risk factors of cardiovascular diseases.

In another study of Kuvin et al.^7^, further confirmed that RHI was negatively associated with risk factors of cardiovascular diseases (r=0.3, P<0.002). The RHI is lower in subjects with a history of hypertension, hyperlipidemia, smoking and CHD. In addition, there is evidence^8^ showing that the increase in blood homocysteine may cause endothelial dysfunction of the coronary artery. Matsuzawa et al.^9^ investigated 240 patients with suspicious CHD who had coronary angiography and Endo-PAT measurement. Results showed RHI in CHD patients was significantly lower than that in patients without CHD (0.51±0.18vs0.70±0.17, P<0.001); in CHD patients, RHI in patients with complex coronary lesions was markedly lower than that in patients with simple coronary lesions (0.48±0.19 vs. 0.58±0.16, P<0.001); patients with coronary lesions at several branches had a dramatically lower RHI when compared with patients with coronary lesions at a single branch (0.51±0.20 vs. 0.43±0.15, P=0.01).

Tycinska et al.^10^ investigated 56 hypertensive patients who were treated with atorvastatin and standard antihypertensive drugs, and drugs were alternated 3 months later. Results showed atorvastatin could further reduce blood pressure, increase NO, reduce endothelin and significantly improve the blood flow mediated vascular dilation. Matsue et al.^11^ investigated 243 CHD patients, the LDL-C of whom increased to ≥70mg/dl after atorvastatin treatment, and they were randomly assigned into 2 groups (10 mg atorvastatin + 10 mg ezetimibe group and 20 mg atorvastatin group). Results showed RHI in patients treated with atorvastatin alone increased markedly when compared with patients treated with atorvastatin and ezetimibe (0.47±0.62 P<0.001vs 0.45±0.48 P=0.399). Atorvastatin can improve the endothelial function, which is independent of LDL-C lowering. Above findings confirm that detection of endothelial function play important roles in the diagnosis and treatment of several diseases.

Endo-PAT 2000 non-invasive diagnostic system is a novel tool used for the detection of endothelial function. Detection is automatic, data are accurate and results may not vary among investigators and can be quantified. This tool may significantly promote the wide application of detection of endothelial function. To early identify coronary lesions which fail to be identified by angiography and timely performed therapies are helpful to reduce the incidence of cardiovascular events in CHD patients and clinically crucial for the therapy and prevention of cardiovascular diseases.
